# Supplementary material for: Drug repurposing for aging research using model organisms
Source: Aging Cell. 2017 Jun 16;16(5):1006–15. doi: 10.1111/acel.12626 (PMC5595691; doi:10.1111/acel.12626)
Supplement: Supplementary file 7 — Data S1 Zip‐Archive of all report cards. [file ACEL-16-1006-s007.zip › RC_PGE.pdf]

PGE

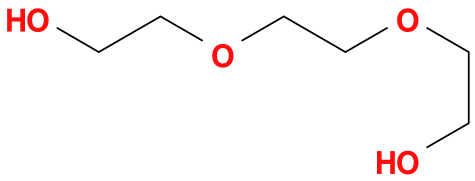

Database identifiers

ChEMBLCompound	CHEMBL1235259
DrugBank	DB02327
CHEBI	44926
ZINC	ZINC01690436
eMolecules	532550

Ranking

	Rank	Score
Drosophila	461/697	0.313
C. elegans	464/591	0.1

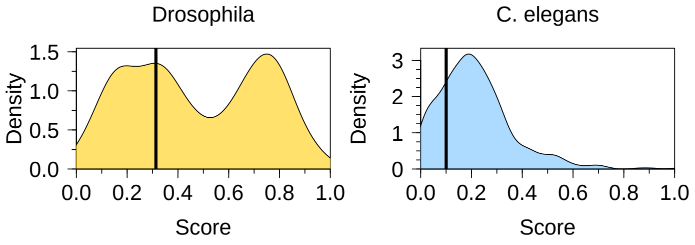

	Ageing implication	Domain conservation	Binding site conservation	Binding affinity	Bioavailability	Lipinski	Promiscuity	Purchasability	Drug approval	**Total**
Drosophila	1.0	0.966	1.0	0.327	(0.9)	0.0	-0.146	0.1	0.075	0.313
C. elegans	1.0	0.946	1.0	0.327	0.231	0.0	-0.146	0.1	0.075	0.1

Names

- **triethylene glycol**
- 1,2-bis(2-hydroxyethoxy)ethane
- 2,2'-ethylenedioxydiethanol
- 2-[2-(2-Hydroxy-Ethoxy)-Ethoxy]-Ethanol
- 3,6-dioxaoctane-1,8-diol
- di-beta-hydroxyethoxyethane
- TEG
- Triethylenglykol
- triglycol

Roles

plasticiser

Status

Approved drug (according to ChEMBL)	No
Classification (according to DrugBank)	experimental
Number of Rule of 5 violations	0
Binding affinity to original target in log units (RF-Score prediction)	4.28
Burns *C. elegans* bioavailability prediction	-3.21

Compound Target Characteristics

Menin

Best gene implication in ageing for this target family came from gene Q8SXU2 annotated in UniProt release 2014_02. Annotation GO 8340 (determination of adult lifespan) was Inferred from Mutant Phenotype

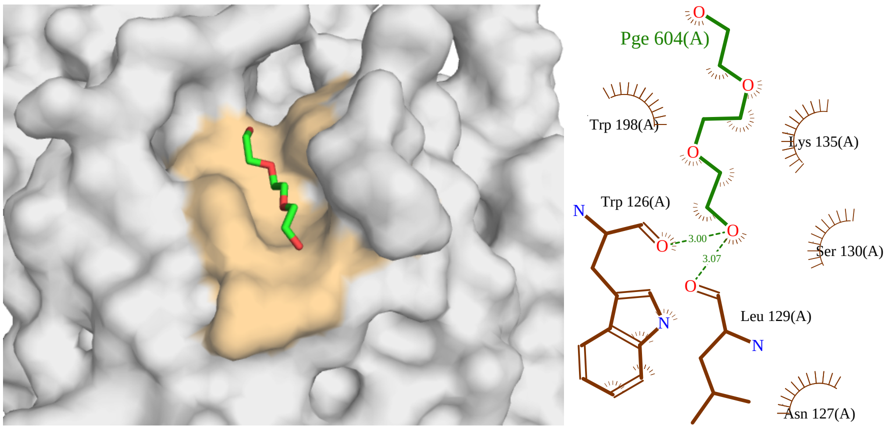

protein	amino acids contacts (binding site)
PDB:4og4:chainA:O00255	W N L S K W F
PDB:4og7:chainA:O00255	W N L S K W F
tr:E7EN32:E7EN32_HUMAN	W N L S K W F
sp:O00255:MEN1_HUMAN	W N L S K W F
sp:Q9WVR8:MEN1_RAT	W N L S K W F
sp:O88559:MEN1_MOUSE	W N L S K W F
tr:F8WHD9:F8WHD9_MOUSE	W N L S K W F
tr:Q8SXU2:Q8SXU2_DROME	W N L I K W L
tr:Q7KXY9:Q7KXY9_DROME	W N L I K W L
tr:Q9VM47:Q9VM47_DROME	W N L I K W L

	whole protein		domain-based		contact-based	
protein	ident	simil	ident	simil	ident	simil
PDB:4og4:chainA:O00255	1.0	1.0	1.0	1.0	1.0	1.0
PDB:4og7:chainA:O00255	1.0	1.0	1.0	1.0	1.0	1.0
tr:E7EN32:E7EN32_HUMAN	0.9	0.9	0.9	0.9	1.0	1.0
sp:O00255:MEN1_HUMAN	1.0	1.0	1.0	1.0	1.0	1.0
sp:Q9WVR8:MEN1_RAT	0.96	0.98	0.96	0.98	1.0	1.0
sp:O88559:MEN1_MOUSE	0.96	0.98	0.96	0.98	1.0	1.0
tr:F8WHD9:F8WHD9_MOUSE	0.95	0.97	0.95	0.97	1.0	1.0
tr:Q8SXU2:Q8SXU2_DROME	0.36	0.64	0.37	0.65	0.71	0.75
tr:Q7KXY9:Q7KXY9_DROME	0.32	0.6	0.34	0.64	0.71	0.75
tr:Q9VM47:Q9VM47_DROME	0.32	0.59	0.34	0.64	0.71	0.75

Mnn1 (FBgn0031885) associated phenotypes

DNA repair defective, chemical sensitive, conditional, heat sensitive, lethal - all die before end of P-stage, melanotic mass phenotype, radiation sensitive, short lived, some die during pharate adult stage, stress response defective

(Information from FlyBase)

Neural cell adhesion molecule 1

Best gene implication in ageing for this target family came from gene F1LUV9 via mapping the annotation from RGD 67378 annotated in RGD 2014-03-11. Annotation GO 7568 (aging) was Inferred from Direct Assay

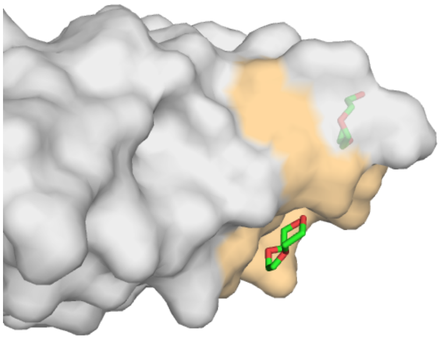

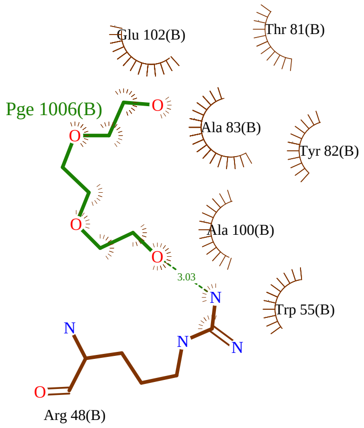

protein	amino acids contacts (binding site)
PDB:2e3v:chainB:P13591	R A V G W K E T T Y A A E
tr:R4GMN9:R4GMN9_HUMAN	R A V G W K E T T Y A A E
tr:H7BYX6:H7BYX6_HUMAN	R A V G W K E T T Y A A E
sp:P13591:NCAM1_HUMAN	R A V G W K E T T Y A A E
tr:S4R389:S4R389_HUMAN	R A V G W K E T T Y A A E
tr:F1LUV9:F1LUV9_RAT	K S L G W K E T R Y A A E
tr:F1LNY3:F1LNY3_RAT	K S L G W K E T R Y A A E
tr:E9Q589:E9Q589_MOUSE	K S L G W K E T R Y S A E
tr:E9QB01:E9QB01_MOUSE	K S L G W K E T R Y S A E
tr:Q058Z9:Q058Z9_DROME	M S D T W E N T T Y L V V
tr:Q9VR25:Q9VR25_DROME	M T E M W E D T V Y L V K
tr:D0Z750:D0Z750_DROME	M S D T W E N T T Y L V V
tr:Q59E14:Q59E14_DROME	M S D T W E N T T Y L V V
tr:Q8T3E5:Q8T3E5_CAEEL	L E E M W T N T Q Y V E S
tr:Q19128:Q19128_CAEEL	L E E M W T N T Q Y V E S
tr:Q8MQ86:Q8MQ86_CAEEL	L E E M W T N T Q Y V E S

	whole protein		domain-based		contact-based	
protein	ident	simil	ident	simil	ident	simil
PDB:2e3v:chainB:P13591	0.98	0.99	0.98	0.99	1.0	1.0
tr:R4GMN9:R4GMN9_HUMAN	0.69	0.7	1.0	1.0	1.0	1.0
tr:H7BYX6:H7BYX6_HUMAN	0.7	0.75	0.58	0.69	1.0	1.0
sp:P13591:NCAM1_HUMAN	1.0	1.0	1.0	1.0	1.0	1.0
tr:S4R389:S4R389_HUMAN	0.92	0.92	1.0	1.0	1.0	1.0
tr:F1LUV9:F1LUV9_RAT	0.92	0.96	0.93	0.97	0.69	0.82
tr:F1LNY3:F1LNY3_RAT	0.94	0.97	0.93	0.98	0.69	0.82
tr:E9Q589:E9Q589_MOUSE	0.78	0.83	0.92	0.97	0.62	0.75
tr:E9QB01:E9QB01_MOUSE	0.94	0.97	0.92	0.97	0.62	0.75
tr:Q058Z9:Q058Z9_DROME	0.08	0.26	0.11	0.35	0.31	0.52
tr:Q9VR25:Q9VR25_DROME	0.1	0.35	0.11	0.38	0.23	0.56
tr:D0Z750:D0Z750_DROME	0.1	0.33	0.11	0.35	0.31	0.52
tr:Q59E14:Q59E14_DROME	0.1	0.33	0.11	0.35	0.31	0.52
tr:Q8T3E5:Q8T3E5_CAEEL	0.17	0.52	0.14	0.46	0.23	0.5
tr:Q19128:Q19128_CAEEL	0.17	0.53	0.14	0.46	0.23	0.5
tr:Q8MQ86:Q8MQ86_CAEEL	0.17	0.51	0.14	0.46	0.23	0.5

FBgn0031627 associated phenotypes

locomotor behavior defective, partially lethal - majority die, song defective

(Information from FlyBase)

Glutamate–cysteine ligase

Best gene implication in ageing for this target family came from gene A4V448 via mapping the annotation from Ensembl FBgn0040319 via mapping the annotation from EntrezGene 53581 via mapping the annotation from GenAgeModels 0293 annotated in GenAge release 17.

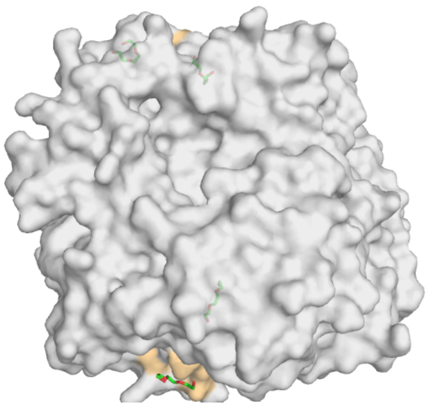

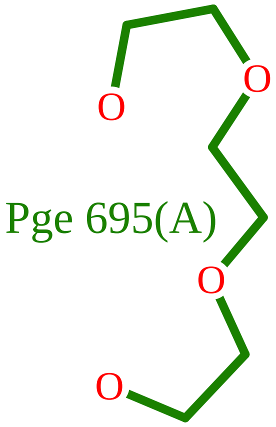

protein	amino acids contacts (binding site)
PDB:3ig5:chainA:P32477	G D F W G P A P G C P D F P W H K L F Y W T Q N N D P R
PDB:3ig8:chainA:P32477	G D F W G P A P G C P D F P W H K L F Y W T Q N N D P R
PDB:3lvv:chainA:P32477	G D F W G P A P G C P D F P W H K L F Y W T Q N N D P R
PDB:3lvw:chainA:P32477	G D F W G P A P G C P D F P W H K L F Y W T Q N N D P R
sp:P48506:GSH1_HUMAN	K H K W G P G P G C P G F P N V E L F F F T Q S S - I R
tr:Q14TF0:Q14TF0_HUMAN	K H K W G P G P G C P G F P N V E L F F F T Q S S - I R
sp:P19468:GSH1_RAT	K H K W G P G P G C P G F P N E E L F F F T Q S S - I R
tr:Q3TEF1:Q3TEF1_MOUSE	K H K W G P G P G C P G F P N E E L F F F T Q S S - I R
sp:P97494:GSH1_MOUSE	K H K W G P G P G C P G F P N E E L F F F T Q S S - I R
tr:A4V448:A4V448_DROME	K Q K W G P G P G A P N F P R E D L Y V - T Q N T - I R
sp:Q9W3K5:GSH1_DROME	K Q K W G P G P G A P N F P R E D L Y V - T Q N T - I R
sp:Q20117:GSH1_CAEEL	K H K W G P G P G V P G F A D K N V F F L T Q N D P I R
tr:E9P9G0:E9P9G0_YEASX	G D F W G P A P G C P D F P W H K L F Y W T Q N N D P R
tr:E9P9M0:E9P9M0_YEASX	G D F W G P A P G C P D F P W H K L F Y W T Q N N D P R
tr:F5CIK1:F5CIK1_YEASX	G D F W G P A P G C P D F P W H K L F Y W T Q N N D P R
sp:P32477:GSH1_YEAST	G D F W G P A P G C P D F P W H K L F Y W T Q N N D P R

protein	whole protein ident	simil	domain-based ident	simil	contact-based ident	simil
PDB:3ig5:chainA:P32477	1.0	1.0	1.0	1.0	1.0	1.0
PDB:3ig8:chainA:P32477	1.0	1.0	1.0	1.0	1.0	1.0
PDB:3lvv:chainA:P32477	1.0	1.0	1.0	1.0	1.0	1.0
PDB:3lvw:chainA:P32477	1.0	1.0	1.0	1.0	1.0	1.0
sp:P48506:GSH1_HUMAN	0.4	0.72	0.42	0.73	0.5	0.59
tr:Q14TF0:Q14TF0_HUMAN	0.4	0.72	0.42	0.73	0.5	0.59
sp:P19468:GSH1_RAT	0.41	0.72	0.42	0.73	0.5	0.6
tr:Q3TEF1:Q3TEF1_MOUSE	0.41	0.72	0.42	0.73	0.5	0.6
sp:P97494:GSH1_MOUSE	0.41	0.72	0.42	0.73	0.5	0.6
tr:A4V448:A4V448_DROME	0.33	0.62	0.35	0.63	0.46	0.52
sp:Q9W3K5:GSH1_DROME	0.33	0.62	0.35	0.63	0.46	0.52
sp:Q20117:GSH1_CAEEL	0.34	0.68	0.36	0.7	0.43	0.57
tr:E9P9G0:E9P9G0_YEASX	1.0	1.0	1.0	1.0	1.0	1.0
tr:E9P9M0:E9P9M0_YEASX	1.0	1.0	1.0	1.0	1.0	1.0
tr:F5CIK1:F5CIK1_YEASX	1.0	1.0	1.0	1.0	1.0	1.0
sp:P32477:GSH1_YEAST	1.0	1.0	1.0	1.0	1.0	1.0

Gclc (FBgn0040319) associated phenotypes

chemical resistant, long lived

(Information from FlyBase)

Gclc (UniProt:Q9W3K5) annotation

Pathway: Sulfur metabolism; glutathione biosynthesis; glutathione from L-cysteine and L-glutamate: step 1/2.

(Information from UniProt)

gcs-1 (UniProt:Q20117) annotation

Pathway: Sulfur metabolism; glutathione biosynthesis; glutathione from L-cysteine and L-glutamate: step 1/2.

(Information from UniProt)

Dual specificity mitogen-activated protein kinase kinase 1

Best gene implication in ageing for this target family came from gene Q02750 annotated in UniProt release 2014_02. Annotation GO subterm of 7568 (aging) was Inferred from Mutant Phenotype

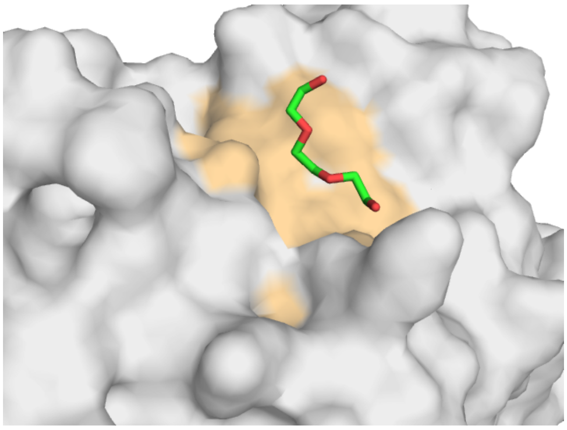

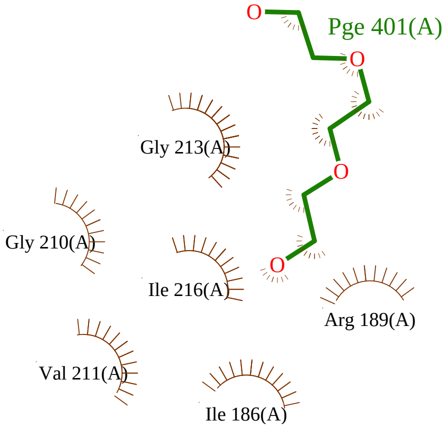

protein	amino acids contacts (binding site)
PDB:3orn:chainA:Q02750	I R G V G I
sp:Q02750:MP2K1_HUMAN	I R G V G I
tr:A4QPA9:A4QPA9_HUMAN	I R G V G I
sp:Q01986:MP2K1_RAT	I R G V G I
tr:Q3TMJ8:Q3TMJ8_MOUSE	I R G V G I
sp:P31938:MP2K1_MOUSE	I R G V G I
sp:Q24324:DSOR1_DROME	I R G V G I
sp:Q10664:MEK2_CAEEL	I R G V G I
sp:P08018:PBS2_YEAST	I R G V G V

	whole protein		domain-based		contact-based	
protein	ident	simil	ident	simil	ident	simil
PDB:3orn:chainA:Q02750	1.0	1.0	1.0	1.0	1.0	1.0
sp:Q02750:MP2K1_HUMAN	1.0	1.0	1.0	1.0	1.0	1.0
tr:A4QPA9:A4QPA9_HUMAN	1.0	1.0	1.0	1.0	1.0	1.0
sp:Q01986:MP2K1_RAT	0.99	1.0	1.0	1.0	1.0	1.0
tr:Q3TMJ8:Q3TMJ8_MOUSE	0.99	1.0	1.0	1.0	1.0	1.0
sp:P31938:MP2K1_MOUSE	0.99	1.0	1.0	1.0	1.0	1.0
sp:Q24324:DSOR1_DROME	0.6	0.82	0.78	0.94	1.0	1.0
sp:Q10664:MEK2_CAEEL	0.48	0.74	0.64	0.89	1.0	1.0
sp:P08018:PBS2_YEAST	0.2	0.36	0.5	0.8	0.83	0.95

Dsor1 (FBgn0010269) associated phenotypes

dominant, lethal - all die before end of pupal stage, neuroanatomy defective, polyphasic, some die during immature adult stage, some die during pupal stage

(Information from FlyBase)

Dsor1 (UniProt:Q24324) annotation

Function: Required downstream of Raf in the sevenless (sev), torso (tor), and Drosophila EGF receptor homolog (DER) signal transduction pathways. Involved in both positive regulation (at the posterior terminus) and negative regulation (at the anterior domain) of tll, as in other terminal class gene products, maybe via the ERK-A kinase. (PubMed:8381718).

Developmental stage: Expressed both maternally and zygotically. (PubMed:8381718).

Ptm: Phosphorylation on Ser/Thr by MAP kinase kinase kinases regulates positively the kinase activity.

(Information from UniProt)

mek-2 (WBGene00003186) associated phenotypes

AWA odorant chemotaxis defective, AWC odorant chemotaxis defective, bacterially unswollen, constipated, germ cell arrest, germ nuclei rachis, lethal, metabolic pathway variant, no oocytes, pachytene region organization variant, pathogen susceptibility increased, rod like larval lethal, sterile, vulval cell lineage variant, vulvaless

(Information from WormBase)

mek-2 (UniProt:Q10664) annotation

Function: Functions in the let-60 Ras signaling pathway; acts downstream of lin-45 raf kinase, but before the sur-1/mpK-1 gene product in controlling vulval cell differentiation.

Enzyme regulation: Activated by tyrosine and threoninephosphorylation catalyzed by MAP kinase kinase kinases.

(Information from UniProt)

Tyrosine-protein phosphatase non-receptor type 11

Best gene implication in ageing for this target family came from gene G5EC24 via mapping the annotation from WormBase WBGene00004214 annotated in WormBase 2014-03-11. Annotation

GO 8340 (determination of adult lifespan) was Inferred from Mutant Phenotype

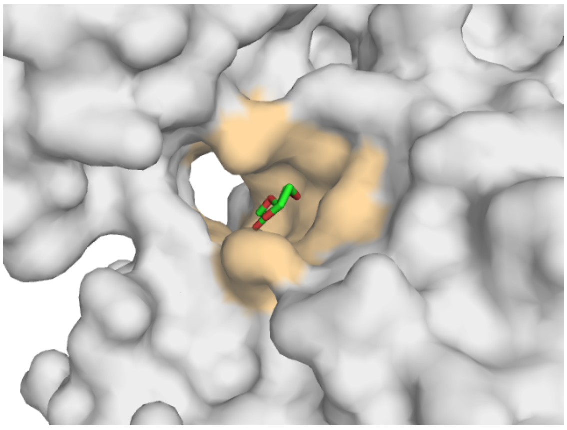

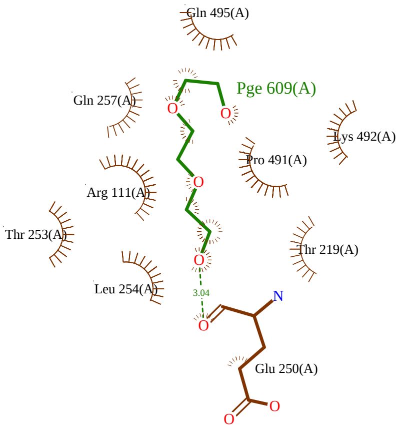

protein	amino acids contacts (binding site)								
PDB:4gwf:chainA:Q06124	R	T	E	T	L	Q	P	K	Q
sp:Q06124:PTN11_HUMAN	R	T	E	T	L	Q	P	K	Q
sp:P41499:PTN11_RAT	R	T	E	T	L	Q	P	K	Q
sp:P35235:PTN11_MOUSE	R	T	E	T	L	Q	P	K	Q
sp:P29349:CSW_DROME	R	T	E	S	L	D	Q	R	Q
tr:G5EC24:G5EC24_CAEEL	R	T	E	R	L	E	Y	K	K

	whole protein		domain-based		contact-based	
protein	ident	simil	ident	simil	ident	simil
PDB:4gwf:chainA:Q06124	1.0	1.0	1.0	1.0	1.0	1.0
sp:Q06124:PTN11_HUMAN	1.0	1.0	1.0	1.0	1.0	1.0
sp:P41499:PTN11_RAT	0.99	1.0	0.99	1.0	1.0	1.0
sp:P35235:PTN11_MOUSE	0.99	1.0	1.0	1.0	1.0	1.0
sp:P29349:CSW_DROME	0.41	0.57	0.45	0.62	0.56	0.74
tr:G5EC24:G5EC24_CAEEL	0.32	0.64	0.36	0.66	0.56	0.69

csw (FBgn0000382) associated phenotypes

dominant, germline clone, heat sensitive, increased cell death, lethal - all die before end of pupal stage, maternal effect, rescuable maternal effect, somatic clone, some die during pupal stage

(Information from FlyBase)

csw (UniProt:P29349) annotation

Function: Required in all receptor tyrosine kinase signaling pathways. Functions downstream of the receptor tyrosine kinase torso, acting in concert with D-Raf via tailless. Also functions downstream of Egfr (epidermal growth factor receptor) and btl (fibroblast growth factor receptor). The SH2 domain suggests that csw effects its role by mediating heteromeric protein interactions. Maternally required for normal determination of cell fates at the termini of the embryo. Required for cell fate specification of the ventral ectoderm, in the developing embryonic CNS and for embryonic tracheal cell migration. Functions during imaginal development for proper formation of adult structures such as eyes, aristae, L5 wing vein and the tarsal claw. (PubMed:1638629, PubMed:8948575).
Subcellular location: Cytoplasm.
Tissue specificity: Expressed uniformly throughout all tissues during embryogenesis. (PubMed:1638629).
Developmental stage: Expressed throughout development. (PubMed:1638629).
Miscellaneous: The PTPase domain is interrupted by a PTPase insert which shares no homologies with other PTPase proteins. This PTPase insert is reminiscent of the kinase insert within the kinase catalytic domains of several receptor tyrosine kinases.

(Information from UniProt)

ptp-2 (UniProt:G5EC24) annotation

Subcellular location: Cytoplasm ECO:0000256—PIRNR:PIRNR000929.

(Information from UniProt)

Tumor suppressor p53-binding protein 1

Best gene implication in ageing for this target family came from gene F6ZRL3 via mapping the annotation from Ensembl ENSMUSG00000043909 via mapping the annotation from EntrezGene 27223 via mapping the annotation from GenAgeModels 0830 annotated in GenAge release 17.

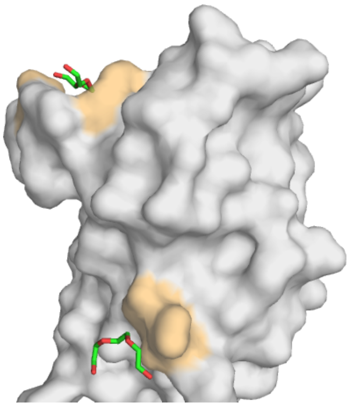

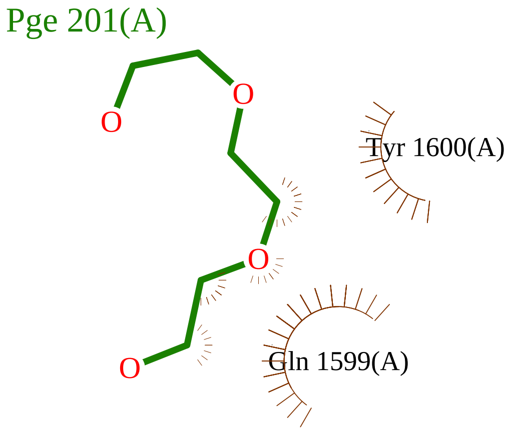

protein	amino acids contacts (binding site)
PDB:3lgf:chainA:Q12888	R E Y W Q Y
PDB:3lgl:chainA:Q12888	R E Y W Q Y
PDB:3lh0:chainA:Q12888	R E Y W Q Y
tr:H7BZY0:H7BZY0_HUMAN	R E Y W Q Y
tr:A6NNK5:A6NNK5_HUMAN	R E Y W Q Y
sp:Q12888:TP53B_HUMAN	R E Y W Q Y
tr:D3ZKQ9:D3ZKQ9_RAT	R E Y W Q Y
tr:F1M9K1:F1M9K1_RAT	R E Y W Q Y
tr:F1M842:F1M842_RAT	R E Y W Q Y
tr:F6ZRL3:F6ZRL3_MOUSE	R E Y W Q Y
tr:Q3V1P7:Q3V1P7_MOUSE	R E Y W Q Y
tr:A2AU89:A2AU89_MOUSE	R E Y W Q Y
tr:A2AU91:A2AU91_MOUSE	R E Y W Q Y
tr:Q7JKP6:Q7JKP6_CAEEL	N S D W V K

	whole protein		domain-based		contact-based	
protein	ident	simil	ident	simil	ident	simil
PDB:3lgf:chainA:Q12888	1.0	1.0	1.0	1.0	1.0	1.0
PDB:3lgl:chainA:Q12888	1.0	1.0	1.0	1.0	1.0	1.0
PDB:3lh0:chainA:Q12888	1.0	1.0	1.0	1.0	1.0	1.0
tr:H7BZY0:H7BZY0_HUMAN	0.08	0.08	1.0	1.0	1.0	1.0
tr:A6NNK5:A6NNK5_HUMAN	0.97	0.97	1.0	1.0	1.0	1.0
sp:Q12888:TP53B_HUMAN	1.0	1.0	1.0	1.0	1.0	1.0
tr:D3ZKQ9:D3ZKQ9_RAT	0.33	0.35	1.0	1.0	1.0	1.0
tr:F1M9K1:F1M9K1_RAT	0.79	0.91	1.0	1.0	1.0	1.0
tr:F1M842:F1M842_RAT	0.81	0.93	1.0	1.0	1.0	1.0
tr:F6ZRL3:F6ZRL3_MOUSE	0.08	0.08	1.0	1.0	1.0	1.0
tr:Q3V1P7:Q3V1P7_MOUSE	0.33	0.34	1.0	1.0	1.0	1.0
tr:A2AU89:A2AU89_MOUSE	0.78	0.9	1.0	1.0	1.0	1.0
tr:A2AU91:A2AU91_MOUSE	0.8	0.92	1.0	1.0	1.0	1.0
tr:Q7JKP6:Q7JKP6_CAEEL	0.11	0.37	0.15	0.57	0.17	0.47

NAD-dependent protein deacetylase sirtuin-2

Best gene implication in ageing for this target family came from gene Q9I7I7 annotated in UniProt release 2014_02. Annotation GO 8340 (determination of adult lifespan) was Inferred from Mutant Phenotype

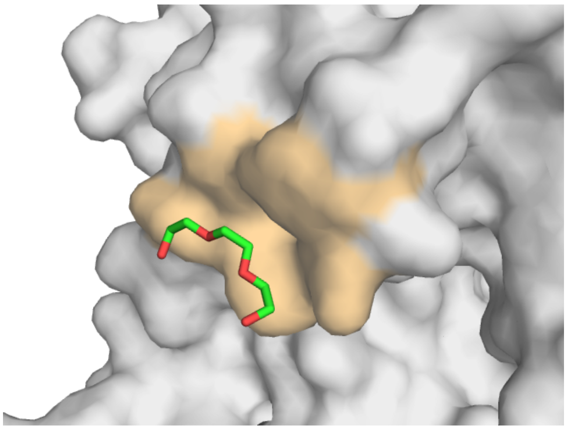

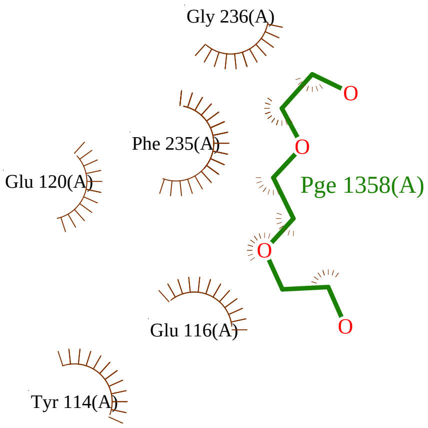

protein	amino acids contacts (binding site)
PDB:3zgo:chainA:Q8IXJ6	Y E E F G
tr:E7EWX6:E7EWX6_HUMAN	Y E E F G
sp:Q8IXJ6:SIR2_HUMAN	Y E E F G
sp:Q5RJQ4:SIR2_RAT	Y E E F G
tr:Q3UJK6:Q3UJK6_MOUSE	Y E E F G
sp:Q8VDQ8:SIR2_MOUSE	Y E E F G
sp:Q9I7I7:SIRT2_DROME	H T D F G
sp:P53686:HST2_YEAST	Y E D F G

	whole protein		domain-based		contact-based	
protein	ident	simil	ident	simil	ident	simil
PDB:3zgo:chainA:Q8IXJ6	0.99	1.0	1.0	1.0	1.0	1.0
tr:E7EWX6:E7EWX6_HUMAN	0.61	0.61	0.68	0.68	1.0	1.0
sp:Q8IXJ6:SIR2_HUMAN	1.0	1.0	1.0	1.0	1.0	1.0
sp:Q5RJQ4:SIR2_RAT	0.78	0.87	0.89	0.97	1.0	1.0
tr:Q3UJK6:Q3UJK6_MOUSE	0.79	0.87	0.89	0.97	1.0	1.0
sp:Q8VDQ8:SIR2_MOUSE	0.88	0.97	0.89	0.97	1.0	1.0
sp:Q9I7I7:SIRT2_DROME	0.43	0.72	0.54	0.83	0.4	0.66
sp:P53686:HST2_YEAST	0.29	0.57	0.38	0.68	0.8	0.9

Sirt2 (FBgn0038788) associated phenotypes
heat sensitive, lethal - all die before end of pupal stage, some die during pupal stage

(Information from FlyBase)

Sirt2 (UniProt:Q9I7I7) annotation
Function: NAD-dependent protein deacetylase (By similarity). May be involved in the regulation of life span. (, PubMed:17159295).
Cofactor: Zn(2+)Note=Binds 1 zinc ion per subunit. ;
Disruption phenotype: Causes lethality during development. Induced silencing shortens life span. (PubMed:17159295).

(Information from UniProt)

NAD-dependent protein deacetylase sirtuin-3, mitochondrial

Best gene implication in ageing for this target family came from gene Q9I7I7 annotated in UniProt release 2014_02. Annotation GO 8340 (determination of adult lifespan) was Inferred from Mutant

Phenotype

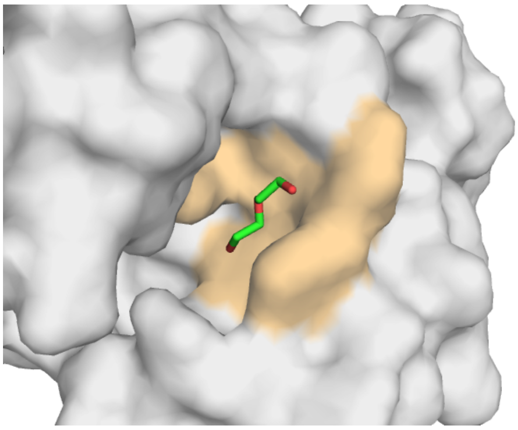

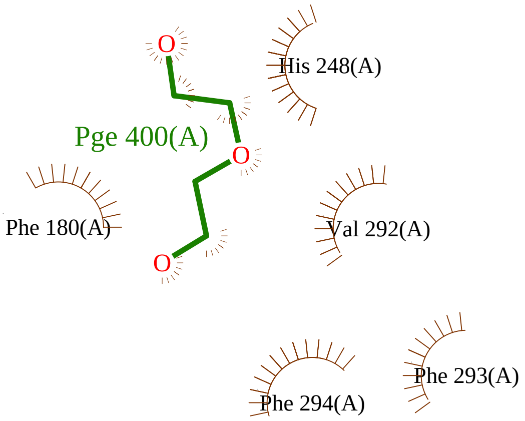

protein	amino acids contacts (binding site)
PDB:3gls:chainA:Q9NTG7	F H V F F G
tr:E9PK80:E9PK80_HUMAN	F H V F F G
tr:E9PN58:E9PN58_HUMAN	F H V F F G
sp:Q9NTG7:SIR3_HUMAN	F H V F F G
tr:B2RZ31:B2RZ31_RAT	F H V F F G
tr:C6ZII9:C6ZII9_RAT	F H V F F G
tr:Q4FJK3:Q4FJK3_MOUSE	F H V F F G
tr:D3YTK6:D3YTK6_MOUSE	F H V F F G
sp:Q8R104:SIR3_MOUSE	F H V F F G
sp:Q9I7I7:SIRT2_DROME	F H V F F G
sp:P53686:HST2_YEAST	F H V F F G

	whole protein		domain-based		contact-based	
protein	ident	simil	ident	simil	ident	simil
PDB:3gls:chainA:Q9NTG7	1.0	1.0	1.0	1.0	1.0	1.0
tr:E9PK80:E9PK80_HUMAN	0.8	0.8	1.0	1.0	1.0	1.0
tr:E9PN58:E9PN58_HUMAN	0.78	0.79	0.93	0.93	1.0	1.0
sp:Q9NTG7:SIR3_HUMAN	1.0	1.0	1.0	1.0	1.0	1.0
tr:B2RZ31:B2RZ31_RAT	0.55	0.62	0.85	0.96	1.0	1.0
tr:C6ZII9:C6ZII9_RAT	0.63	0.74	0.85	0.96	1.0	1.0
tr:Q4FJK3:Q4FJK3_MOUSE	0.55	0.62	0.85	0.96	1.0	1.0
tr:D3YTK6:D3YTK6_MOUSE	0.48	0.55	0.61	0.67	1.0	1.0
sp:Q8R104:SIR3_MOUSE	0.62	0.72	0.85	0.96	1.0	1.0
sp:Q9I7I7:SIRT2_DROME	0.34	0.55	0.43	0.68	1.0	1.0
sp:P53686:HST2_YEAST	0.26	0.49	0.33	0.61	1.0	1.0

Sirt2 (FBgn0038788) associated phenotypes

heat sensitive, lethal - all die before end of pupal stage, some die during pupal stage

(Information from FlyBase)

Sirt2 (UniProt:Q9I7I7) annotation

Function: NAD-dependent protein deacetylase (By similarity). May be involved in the regulation of life span. (, PubMed:17159295).

Cofactor: Zn(2+)Note=Binds 1 zinc ion per subunit. ;

Disruption phenotype: Causes lethality during development. Induced silencing shortens life span. (PubMed:17159295).

(Information from UniProt)
